# A study on the codon usage bias of arenavirus common genes

**DOI:** 10.3389/fmicb.2024.1490076

**Published:** 2025-01-23

**Authors:** Pablo Daniel Thomas, María Florencia Ferrer, Mauricio J. Lozano, Ricardo Martín Gómez

**Affiliations:** ^1^Laboratorio de Patogénesis Viral, Instituto de Biotecnología y Biología Molecular, CONICET-UNLP, La Plata, Argentina; ^2^Laboratorio de Genómica y Ecología Molecular de Microorganismos del Suelo Asociados con Plantas, Instituto de Biotecnología y Biología Molecular, CONICET-UNLP, La Plata, Argentina

**Keywords:** *Arenaviridae*, codon usage, phylogeny, *Mammarenavirus*, evolution

## Abstract

**Introduction:**

The *Arenaviridae* family consists of the genera *Mammarenavirus*, *Reptarenavirus*, *Hartmanivirus*, *Antennavirus* and *Innmovirus*. The codon usage bias between the different genera has not yet been studied comparatively.

**Methods:**

We retrieved the arenavirus genome sequences from public databases and used bioinformatics tools to compare the codon usage bias between the different genera for the GPC, NP and L proteins, common to all arenaviruses.

**Results and discussion:**

Hartmaniviruses show a larger codon usage bias, which can be partially explained by mutational bias. Patterns of relative use of synonymous codons were maintained within genera, with individual genera differing in their preference for the third nucleotide position in synonymous codons. Of the three proteins examined, the ARN polymerase L protein exhibited a slightly stronger codon usage bias, but overall, the patterns were repeated between genera for the three proteins examined. Our results suggest that codon usage pattern bias in arenaviruses is influenced by selection pressure and to a lesser extent by mutational selection.

## Introduction

The *Arenaviridae* family is formed by the genera *Mammarenavirus, Reptarenavirus, Hartmanivirus, Antennavirus* and *Innmovirus* (Radoshitzky et al., 2023). The mammarenaviruses, as the name suggests, infect mammals, mostly rodents, and their geographical distribution is related to their natural reservoirs (Salazar-Bravo et al., 2002; Gonzalez et al., 2007). The genus has been divided into two groups based on its place of origin: Old World (OW) and New World (NW) mammarenaviruses. The former are mainly found in Africa, while the latter are native to the Americas. Some members of the *Mammarenavirus* genus can infect humans and cause hemorrhagic fever. Examples are the OW viruses Lassa (LASV) and Lujo (LUJV) in West and South Africa (Emonet et al., 2006) and the NW viruses Junin (JUNV), Machupo (MACV), Chapare (CHPV), Guaranito (GTOV) and Sabia (SABV) in South America (Sarute and Ross, 2017). The genera *Reptarenavirus* and *Hartmanivirus* have been found in snakes (Hepojoki et al., 2015b), and some members of the genus *Reptarenavirus* cause Boid Inclusion Body Disease (BIBD) in captive snakes (Stenglein et al., 2012; Hetzel et al., 2013). Antennaviruses, for their part, infect striated frogfish and salmon (Shi et al., 2018; Mordecai et al., 2019), while *Innmovirus* is the only genus whose natural reservoir is still unknown (Chen et al., 2022; Chen et al., 2023).

Arenaviruses are single-stranded ambisense RNA viruses with some differences between the genera. Mammarenaviruses and reptarenaviruses have a bisegmented RNA with an ambisense coding strategy for 4 proteins: GPC and NP are coded in the S segment and L and Z in the L segment. Hartmaniviruses have a bisegmented RNA with an ambisense S segment coding for the GPC and NP proteins and a negative-sense RNA L segment coding for the L protein, but they have no homolog for the Z protein of mammarenavirus and reptarenavirus. Antennaviruses have genomes consisting of three genomic segments, a negative-sense S segment that encodes NP, an ambisense segment that encodes GPC and an unknown protein, and a negative-sense L segment that encodes the L protein but also has no homologous Z protein. Finally, Innmovirus has three negative-sense RNA segments, the S segment that encodes NP, the intermediate segment that encodes GPC and an unknown protein, and the L segment that encodes the L protein (Hepojoki et al., 2015b; Shi et al., 2018; Chen et al., 2022; Radoshitzky et al., 2023).

The genetic code consists of 64 codons, 61 of which code for amino acids and 3 for stop signals during protein synthesis. Since there are only 20 common amino acids, the genetic code is degenerate, i.e., several codons – synonymous codons – are translated into the same amino acids, with the exception of methionine and tryptophan. However, not all synonymous codons are used with the same frequency, with some organisms favoring some codons over others. This unequal use of codons is referred to as codon usage bias or pattern (Ikemura, 1981; Plotkin and Kudla, 2011; Chaney and Clark, 2015). Codon usage bias is a critical measure of genome evolution and has been found in most organisms, from prokaryotes to eukaryotes and viruses (Sharp and Li, 1987; Plotkin and Kudla, 2011; Belalov and Lukashev, 2013; Chaney and Clark, 2015). Several factors have been found to influence biased codon selection, including gene expression levels, translation, base mutations, genetic drift, transcription factors and the external environment (Bulmer, 1991; Butt et al., 2016; Velazquez-Salinas et al., 2016), with natural selection and mutational pressure in combination with genetic drift being the most important factors (Belalov and Lukashev, 2013).

In viral genomes, codon usage is a driving force for their evolution (Dutta et al., 2020). Some researchers suggest that mutational biases are the primary determinant of codon usage patterns in human RNA viruses (Jenkins and Holmes, 2003; Nasrullah et al., 2015; van Hemert et al., 2016; Tort et al., 2020), while others have identified the dominant influence of natural selection (Wang et al., 2016; Baha et al., 2019; Khandia et al., 2019; Luo et al., 2020). As parasitic organisms, viruses have some features in their genomes that differ from those of prokaryotes and eukaryotes, e.g., they rely on the translational machinery of their hosts for gene expression. This interaction between virus and host influences overall viral survival, adaptation, evasion of the host immune response and evolution (Nasrullah et al., 2015; Rahman et al., 2018; Nguyen et al., 2021).

Relevant human RNA viruses that have already been analyzed for codon usage bias include orthomyxoviruses (Luo et al., 2019), flaviviruses (Jenkins et al., 2001), lyssaviruses (Zhang et al., 2018), bornaviruses (He et al., 2014), polioviruses (Zhang et al., 2011), retroviruses (RoyChoudhury and Mukherjee, 2013) and coronaviruses (Mogro et al., 2022), among others. In contrast, research on arenaviruses is limited to one study on codon adaptation index (CAI) (Sharp and Li, 1987) for OW LASV and LCMV (Pontremoli et al., 2017) and one study on codon usage bias in NW CHPV and SABV (Malhotra and Kumar, 2021).

In this study, we performed an analysis of codon usage bias in the *Arenaviridae* family, focusing on the three proteins that are common to all members. We used different indices such as Effective Number Codons (ENC) (Wright, 1990), Relative Synonymous Codon Usage (RSCU) and CAI to draw general conclusions that could improve the understanding of the evolutionary pressures controlling the evolution of arenaviruses and their adaptability to different hosts and environments.

## Materials and methods

### Genomic sequences

All available arenaviruses with complete coding sequences of the three proteins that are common to all members (GPC, NP, and L proteins) were collected and downloaded from the nucleotide database of the National Center for Biotechnology (NCBI, GenBank)^1^ using the reference species listed in the ICTV report for the *Arenaviridae* family (Radoshitzky et al., 2023). The list of virus names, abbreviations, accession numbers and host information can be found in Supplementary Tables 1, 2.

Human coding sequences for constitutive genes that are highly expressed in all tissues were obtained from the Human Protein Atlas^2^. For this purpose, a table of all proteins constitutively expressed in all human tissues was downloaded, the 500 most highly expressed proteins were selected, and their coding sequences were obtained from the protein-coding transcripts of the human genome v.38^3^ using custom Python scripts. For further comparative analysis with lowly expressed constitutive genes in all human tissues, a table of median transcripts per million (TPM) in all tissues (2017/06/05, v8 RNASeqCv1.1.9) was downloaded from GTEx Portal^4^. From this table, Ensembl IDs were extracted where the TPM was non-zero in all tissues, the maximum value did not deviate significantly from the group variance, and the lowest medians were identified. Protein-coding transcripts for the 500 least-expressed human proteins were then obtained from Ensembl release 112 (Harrison et al., 2024) using additional custom Python scripts.

Host coding sequences were obtained from the NCBI nucleotide database for the snake family *Boidae* (txid: 8572) and the rodent families *Muridae* (txid: 10066) and *Cricetidae* (txid: 337677). Due to the lack of coding sequences for *Antennariidae* (txid: 241819) and the fact that two of the four antennaviruses were found in sockeye and chinook salmon (*Oncorhynchus nerka* and *Oncorhynchus tshawytscha*, respectively), the coding sequences for the family *Salmonidae* (txid: 8015) were chosen instead.

### Phylogenetic analysis

The coding sequences for the L and NP proteins of all selected arenaviruses were aligned and concatenated using MAFFT v7 software (Katoh et al., 2019) with default settings. The resulting multiple sequence alignment was used to generate a maximum likelihood phylogenetic tree using IqTree (Trifinopoulos et al., 2016). Default settings were used, and the best fitting model was LG + F + I + G4.

### Analysis of synonymous codon usage

The guanidine-cytosine (GC) composition for all viral coding sequences was calculated using the online software CAIcal^5^ (Puigbo et al., 2008). This software was also used to analyze the nucleotide composition of the viral sequences and for RSCU and CAI calculations as well as for GC in the third codon position (GC3). ENC was calculated with R scripts using coRdon R software (Elek et al., 2018). Correspondence analysis was performed with the R package FactoMineR (Husson et al., 2008) using the previously calculated RSCU values and presented with the R package factoextra (Kassambara and Mundt, 2020). The R scripts were processed with the software RStudio build 369 23.12.0 (RStudioTeam, 2020).

### Statistical analysis

Most of the results were presented as a violin plot with jitter points (min-max, the horizontal upper, lower bottom and middle lines indicate the 75th percentile or upper quartile, the median and the 25th percentile or lower quartile). The Shapiro–Wilk normality test was applied to the data to decide whether a parametric ordinary one-way analysis of variance (ANOVA) or a non-parametric one-way analysis of variance (ANOVA) (Kruskal–Wallis) should be performed. The results are then followed by either the Tukey, Dunn or Mann–Whitney multiple comparison test to determine significant differences between the groups for parametric or non-parametric analyses. In all cases, *p* values of less than 0.05 were considered statistically significant. Almost all statistical analyses were performed using Prism 8 software^6^. Statistical analysis of the RSCU was performed using custom Python scripts.

## Results

### Phylogenetic analysis

We constructed a phylogenetic tree of the *Arenaviridae* family using maximum likelihood and concatenated amino acid sequence alignments of the NP and L proteins of members with fully sequenced genomes. The results showed a tree that has a similar topology to the tree hosted at ICTV using only L (Radoshitzky et al., 2019), although it has some differences, particularly with respect to the phylogenetic distance of the NW mammarenaviruses and reptarenaviruses and the inclusion of newly sequenced genomes (Supplementary Figure S1).

### Compositional analysis

**Figure 1:**
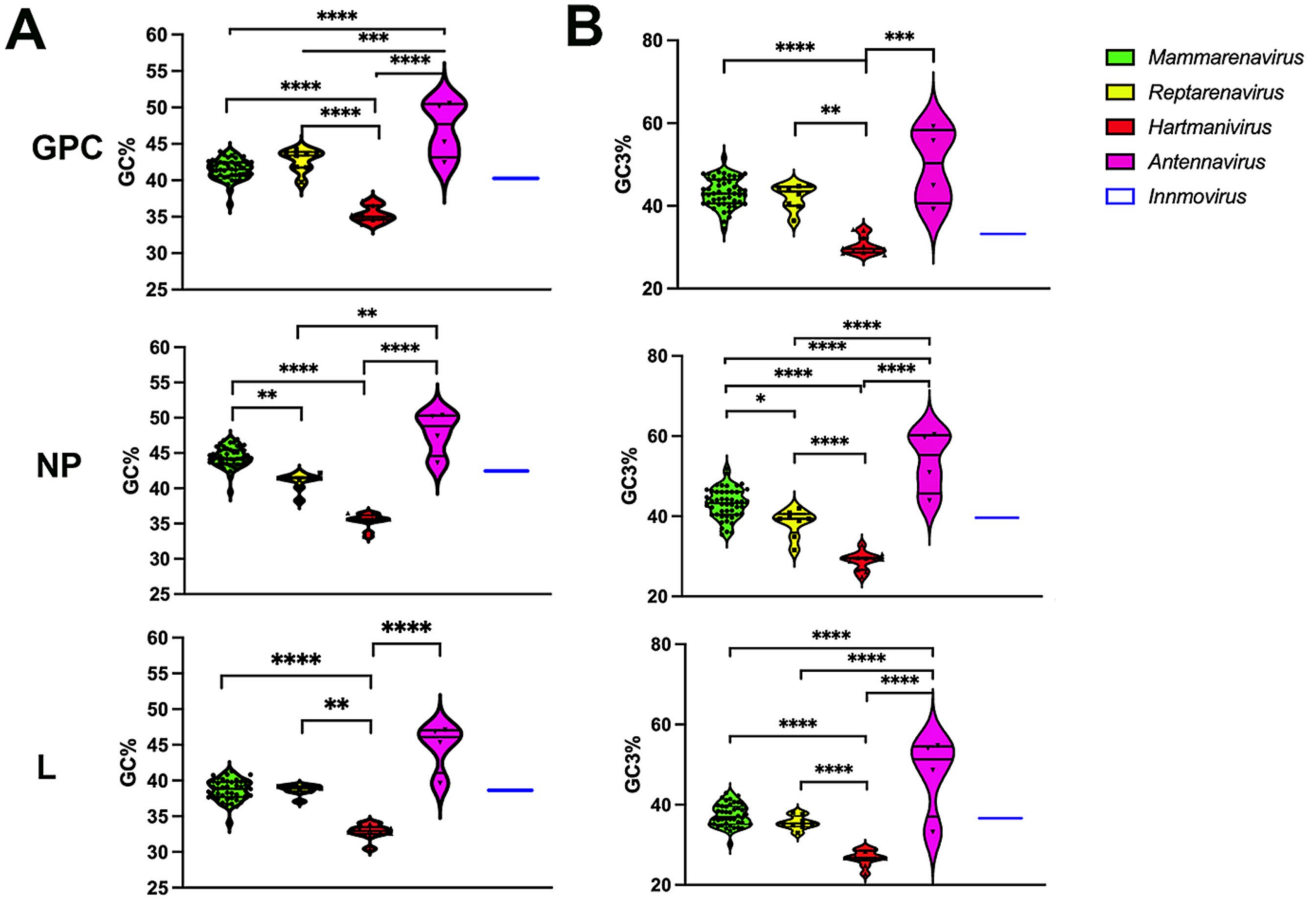
**(A)** Analysis of the guanine-cytosine composition of arenavirus genera for GPC, NP and L protein sequences. A violin plot shows the GC percentage for the coding sequences of the *GPC*, *NP* and *L* genes of each *Arenavirus* genus. The middle horizontal line represents the median GC proportion within the genus while top and bottom horizontal lines represent upper and lower quartile, respectively. *p* values were calculated using either Tukey’s test (GPC) or Dunn’s test (L and NP) for multiple comparisons based on the normality distribution of the data (p* < 0.05, p** < 0.01, p*** < 0.001, p**** < 0.0001). **(B)** Guanine-cytosine composition at the third codon position of arenavirus genera for GPC, NP and L protein sequences. A violin plot showing the GC percentage at the third codon position for the coding sequences of the *GPC*, *NP* and *L* genes of each *Arenavirus* genus. *p*-values were calculated using either Tukey’s test (NP and L) or Dunn’s test (GPC) for multiple comparisons based on the normality distribution of the data (p* < 0.05, p** < 0.01, p*** < 0.001, p**** < 0.0001).

The GC% content of *GPC*, *NP* and *L* genes was below 50 for all arenaviruses, with the lowest values for *Hartmanivirus* at 30/35% and the highest for *Antennavirus* at 45/50% (Figure 1A). These values are lower than those of the coding sequences of known hosts, namely 52.3% for *Homo sapiens*, 51.8% for *Mus musculus* and *Rattus norvegicus* (Zhang et al., 2004). For snakes and salmonids, only the genomic GC content could be analyzed, which is 42% for *Charina bottae* (Grismer et al., 2022) and 43.5% for *O. nerka* (Christensen et al., 2020). These values are similar to the 42% of *R. norvegicus* and *M. musculus* and the 41% of *H. sapiens*. A similar trend can be observed for the GC composition at the third codon position, but with higher overall values, ranging from 30 to almost 60%, depending on the gene and species analyzed (Figure 1B).

### Preferred codons

The RSCU for the *GPC*, *NP* and *L* genes of the analyzed arenaviruses are listed in Table 1.

**Table 1:** Relative synonymous codon usage for arenaviral common proteins, averaged per genus and family.

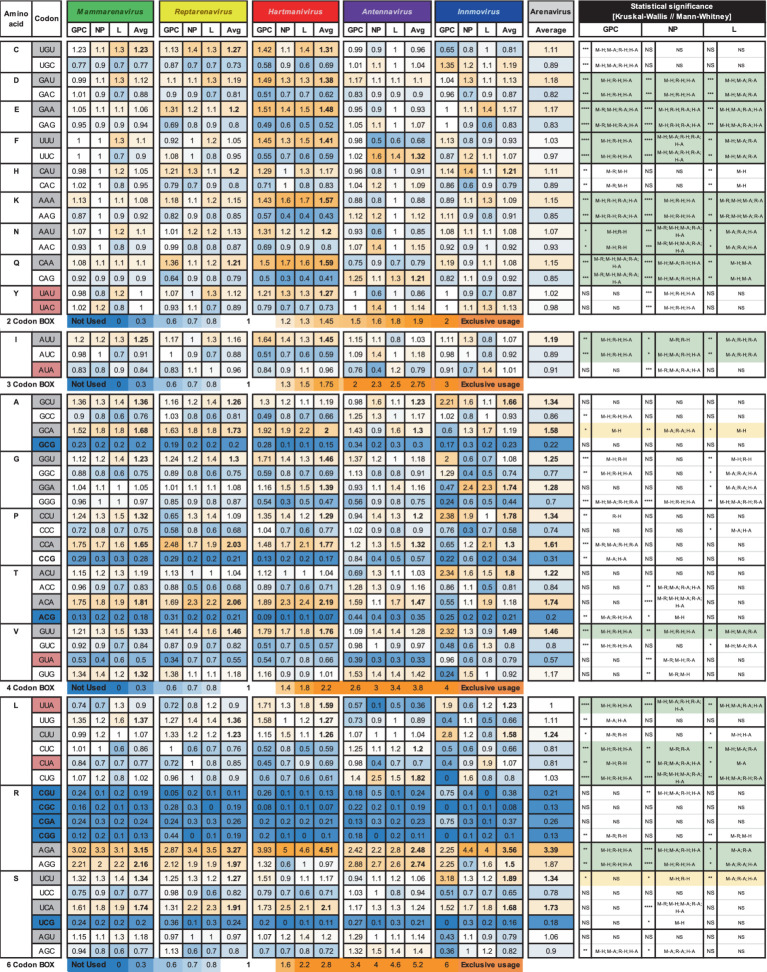

CG containing codons are highlighted in blue, and the TA containing ones in light red. RSCU values greater than 1.2 are represented with bold characters. On the *Arenavirus* column, codons with a general usage of more than 1.2 are in bold characters. The color scale from blue to orange is different for each codon box as is the maximum RSCU value (Indicated as *n* codon Box). In the statistical analysis table (left), ****, ***, **, and * corresponds to Kruskal-Wallis test *p*-values <0.0001, <0.001, <0.01, and < 0.05, respectively. Mann–Whitney test was used for pairwise comparisons, where M, Mammarenavirus; R, Reptarenavirus; H, Hartmanivirus; A, Antennavirus; I, Innmovirus. All significant values with *p* < 0.05 were considered. Green shading highlights a significant difference in the Kruskal-Wallis test for all proteins.

RSCU values above 1.0 refer to codons that were used more frequently, and values below 1.0 refer to codons that were used less frequently, while a value equal to 1.0 means that all codons were used equally frequently (Sharp and Li, 1987). We observed a general preference for codons with A or U in the third position among the synonymous codons, with 9 amino acids (I, A, G, P, T, V, L, R, and S) having an RSCU value of >1.2 and the rest having either a similar usage (~ 1) or a weaker preference (> 1 but <1.2) in most genera (GC3 < 50, Figure 1B and Table 1), with the exception of *Antennavirus*, where only amino acids A, P, T and R showed a preference for codons with A/U terminus. *Hartmanivirus*, on the other hand, showed significantly higher RSCU values for most codons with A/U terminus compared to the other genera, even in the 2 codon boxes (Pairwise significant differences in *Hartmanivirus*, which have a *p*-value <0.05, are labeled as M-H, R-H and H-A). In the case of arginine, codons AGA and AGG were generally more represented in all genera than codons in the CG box, which were underrepresented. The statistically significant differences between genera varied between proteins for some codons, with cases in which there were significant differences for only one protein, such as for NP for codons UAU, UAC, ACC, ACA, and CGU; and for GPC for UGU, UGC, CAU, CAC, GCC, CCU, CCA, CCG, and UUG, among others. In addition, all codons with CG or UA dinucleotides in their sequence and certain G-start and C-end codons – which can form CG depending on the codon pair – were underrepresented. RSCU values tended to be similar between the different proteins, with the exception of *Innmovirus*, where certain codons were overrepresented in some proteins and underrepresented in others, due to insufficient sample size.

### Effective number of codons

**Figure 2:**
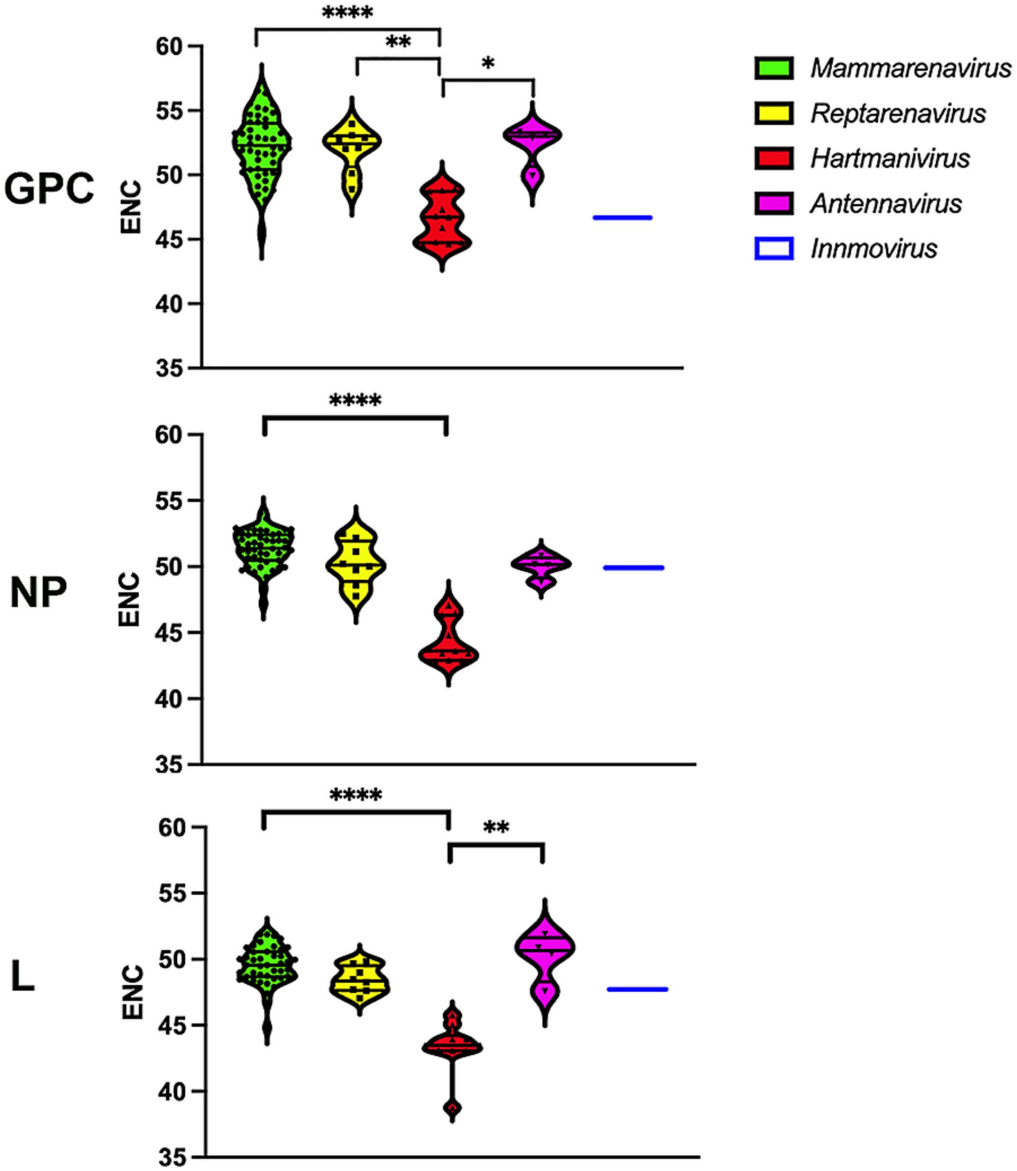
Differences in ENC between arenavirus genera for GPC, NP and L protein sequences. A violin plot shows the ENC for the coding sequences of the *GPC*, *NP* and *L* genes of each arenavirus genus. *p*-values were calculated in all cases using Dunn’s test for multiple comparisons based on the normality distribution of the data (p* < 0.05, p** < 0.01, p*** < 0.001, p**** < 0.0001).

The values for the effective number of codons (ENC) range from 20, indicating an extreme bias in the use of codons, as only one codon is used for each amino acid, to 61, indicating that there is no preference and that all possible synonymous codons are used equally (Wright, 1990). We determined the ENC to assess the extent of codon usage bias (Figure 2). A slight codon usage bias was observed for most genera, with ENC values close to 50 for all proteins analyzed. Remarkably, *Hartmanivirus* showed a significantly lower ENC value (~45) for all proteins, which could be partly explained by the more biased nucleotide composition (Figure 1).

**Figure 3:**
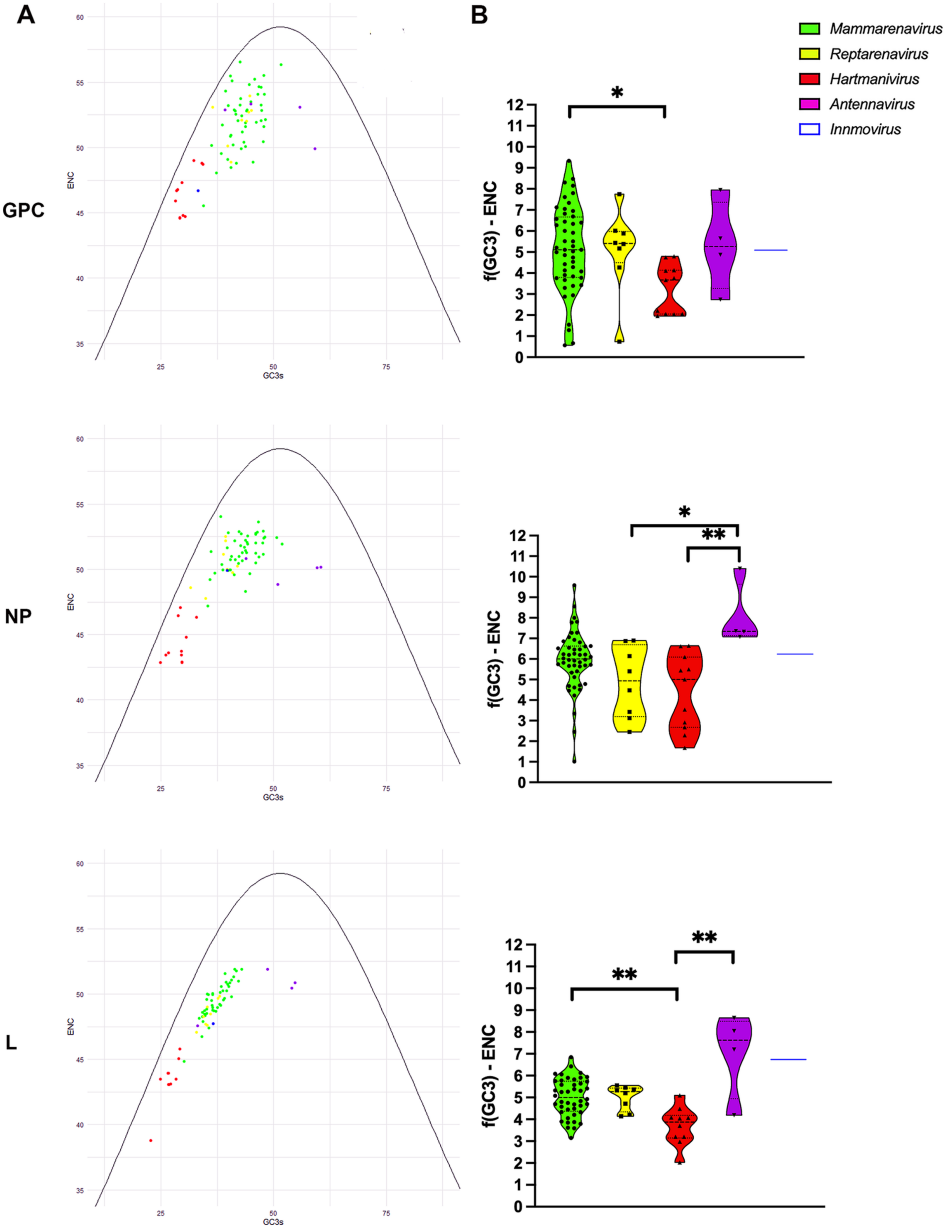
ENC-plot analysis of arenavirus genera for GPC, NP and L protein sequences. **(A)** The continuous purple curve represents the expected ENC values when the GC3 composition only restricts codon usage bias (no selection). The dots represent the ENC values for each of the coding sequences of all analyzed viruses. Different colors indicate the genera as shown. The color coding is the same in all plots. **(B)** Difference between the ENC values and the expected ENC values under the hypothesis of no selection. A Kruskal-Wallis test followed by a Dunn’s test was used to compare the data between genera. * and ** correspond to *p*-values <0.05 and 0.01, respectively.

To further investigate the effects of mutational pressure on patterns of codon usage, we generated an ENC plot. Arenavirus genes where codon selection is only affected by mutational pressure fall on or slightly below the expected ENC curve assuming no natural selection calculated as f(GC3) (dos Reis et al., 2004), with the effect of selection on codon usage being greater the further the points are from the curve. We found that all points corresponding to proteins from the different species were below the curve for the expected ENC value for each GC3, as suggested by other researchers (Wright, 1990), with *Mammarenavirus* and *Antennavirus* being the furthest apart (Figures 3A,B). In addition, GPC and NP were further apart than L, whose points were visibly closer to the curve. The difference between the ENC values and the expected ENC values under the hypothesis of no selection *f*(GC3) was compared between genera for each protein (Figure 3B). While mammarenaviruses and reptarenaviruses had the same median *f*(GC3) ENC value between 5 and 6 for all three proteins, antennaviruses and hartmaniviruses differed significantly from each other in NP and L, with the former having higher median value than the other genera (*f*(GC3) ENC value >7). These results suggest that factors other than mutational pressure, including natural selection, influence the evolution of codon usage in arenaviruses, and that this effect is more pronounced in antennaviruses and in L and NP when compared to GPC proteins. In contrast, the results for *Hartmanivirus* indicate a relatively stronger influence of mutational pressure in this genus.

**Figure 4:**
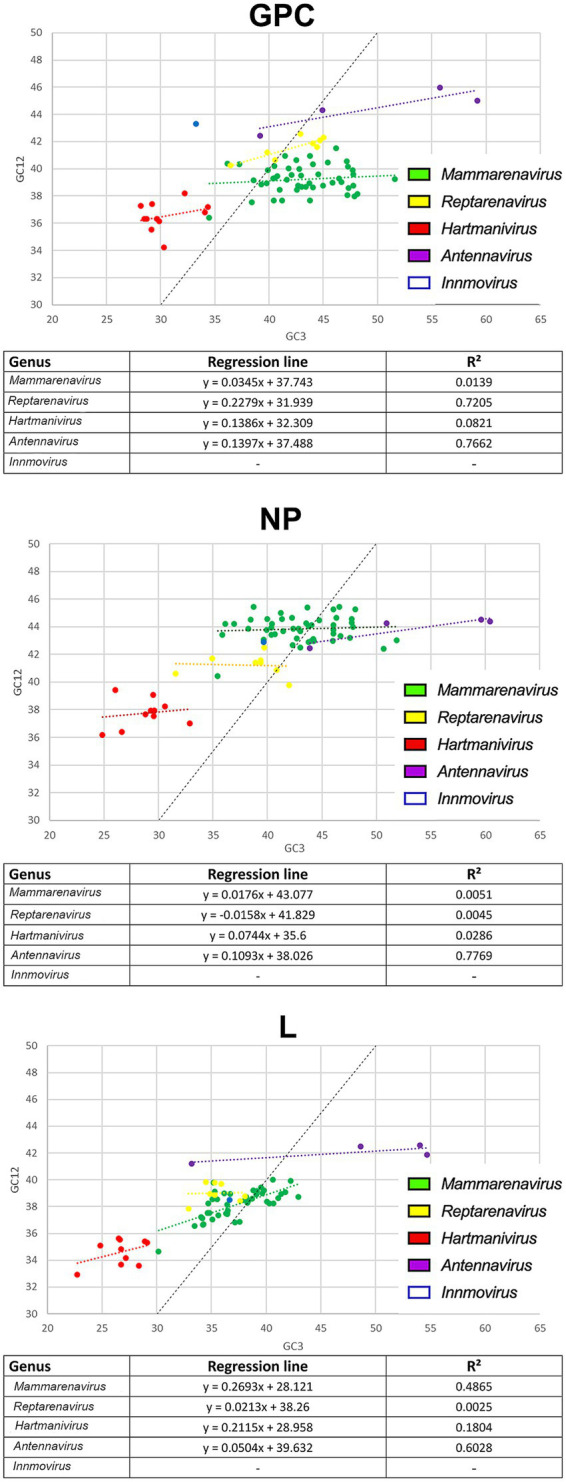
Neutrality plot analysis of arenavirus genera for GPC, NP and L protein sequences. Each genus is shown in a different color. The formula and R2 for the regression line plotted for each genus are indicated below each image.

To further investigate the effects of natural selection on arenaviruses, we constructed neutrality plots for *GPC*, *NP* and *L* protein genes by genus, in which mutational pressure and natural selection pressure are expressed as slopes of the regression line (Nasrullah et al., 2015). We observed an important role of natural selection in shaping codon usage in arenaviruses (Figure 4). In all genera, natural selection explains more than 73% (although in most cases no correlation was found between GC12 and GC3). The genera for which mutational pressure played a greater role were: *Reptarenavirus* and *Antennavirus* GPC with 22 and 14% respectively, *Antennavirus* NP with 11% and *Mammarenavirus* and *Hartmanivirus* L with 27 and 21%, respectively.

### Comparative host adaptability

**Figure 5:**
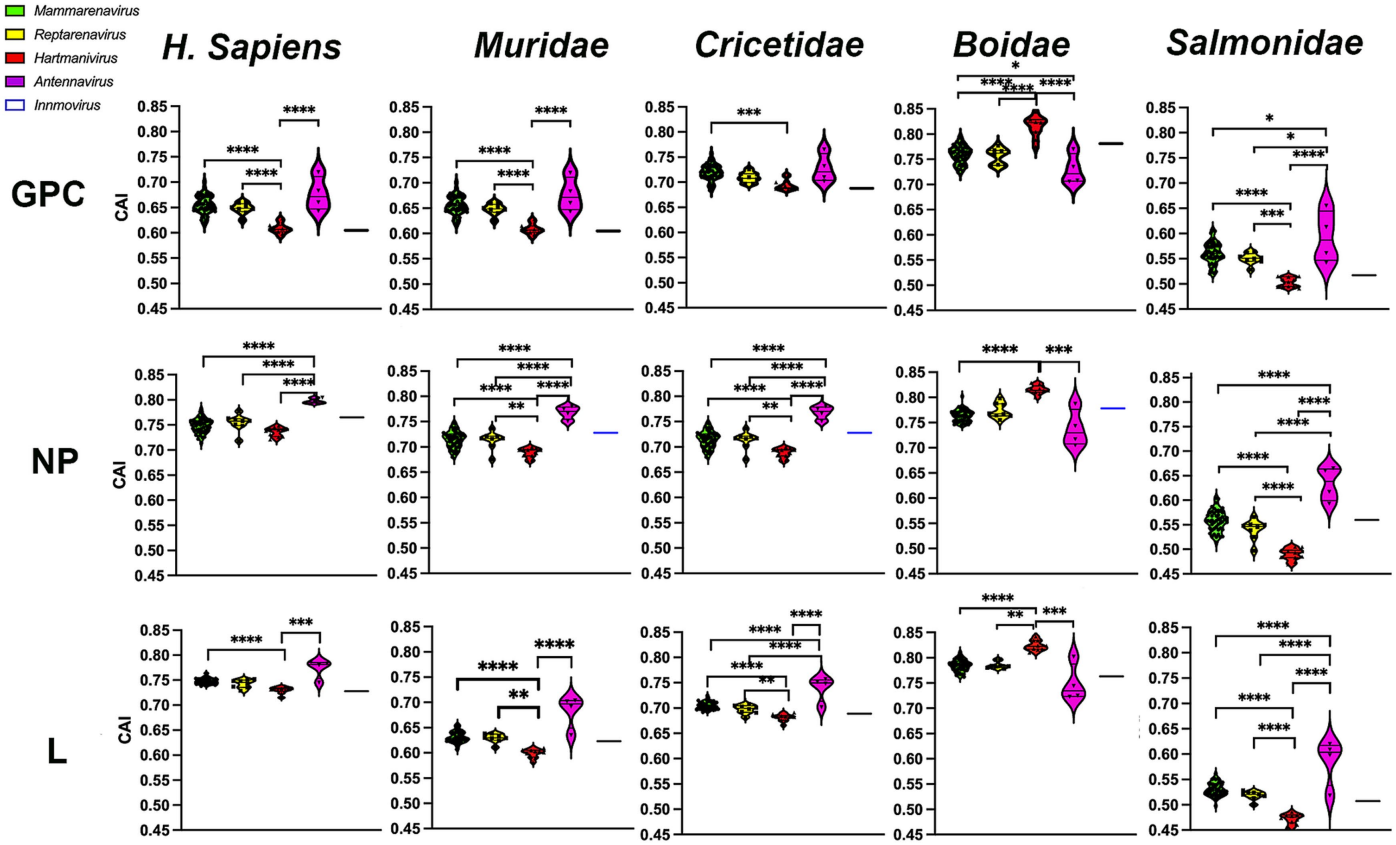
Codon adaptation indices of arenavirus genera for GPC, NP and L protein sequences in different hosts. CAI values were calculated using codon usage tables obtained from sequences of *H. sapiens* and four different taxonomic animal families. *p*-values were calculated using Tukey or Dunn tests for multiple comparisons based on the normality distribution of the data (p* < 0.05, p** < 0.01, p*** < 0.001, p**** < 0.0001).

To investigate whether the observed selection favors optimal codons and thus rapid and efficient translation of viral proteins in the host, we next calculated the CAI (Sharp and Li, 1987) using the CDS of all human genes and the CDS of their natural hosts, the families *Boidae, Muridae, Cricetidae, and Salmonidae*. CAI values range from 0 to 1, with higher CAI values indicating higher expression levels and greater adaptation to the host (Sharp and Li, 1987). Our results show that CAI levels were highest in *H. sapiens* and the *Boidae* family and lowest in the *Salmonidae* family (Figure 5). It was expected that *Mammarenavirus* genes in the family *Muridae* would have lower CAI values than those in the family *Cricetidae*, since most of their natural hosts belong to the latter. It was also expected that *Hartmanivirus* and *Antennavirus* would have higher CAI values in the family *Boidae* and *Salmonidae*, respectively, as these are their natural hosts. However, there were also unexpected results, including the fact that: (a) *Mammarenavirus* proteins had higher CAI values in *H. sapiens* than in the family *Cricetidae*; (b) *Antennavirus* had equal or significantly higher CAI values than mammarenaviruses for the three proteins in *H. sapiens*, the family *Muridae* and the family *Cricetidae*; (c) with the exception of *H. sapiens*, *Reptarenavirus* and *Hartmanivirus* generally showed significant differences between them, even in the family *Boidae*, the natural host of both genera; and finally (d) the high similarity between *Mammarenavirus* and *Reptarenavirus* in all hosts.

**Figure 6:**
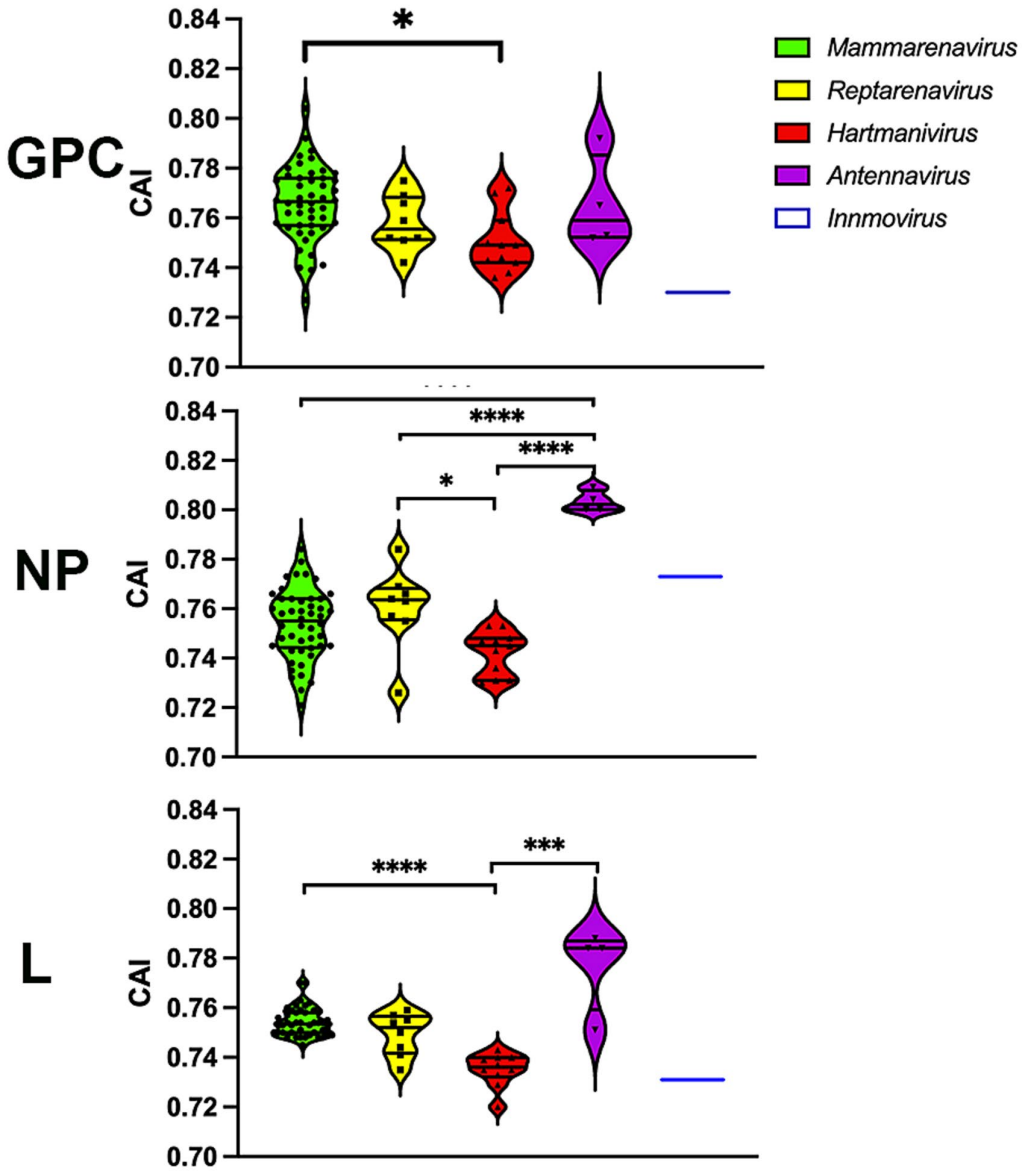
CAI calculated using the 500 most highly constitutively expressed human protein sequences as reference set. *p*-values were calculated using either Tukey’s (NP and GPC) or Dunn’s (L) tests for multiple comparisons based on the normality distribution of the data (p* < 0.05, p** < 0.01, p*** < 0.001, p**** < 0.0001).

To further investigate whether codon usage bias tends to be optimally expressed, we performed a CAI calculation with 500 of the most highly expressed proteins in all human tissues (Uhlen et al., 2015), so that higher CAI_HHE_ (HHE: human highly expressed) values could include more accurate and faster translation, in addition to several factors. The results show that most arenavirus proteins have CAI_HHE_ values in the range of 0.7–0.82 (Figure 6), which are similar to those of human proteins (CAI_HHE_ ~ 0.78 on average), regardless of whether they are highly or lowly expressed (HLE: human lowly expressed) (the mean CAI_HHE_ values for HHE and HLE are 0.781 and 0.782 respectively, and are not significantly different in the Mann–Whitney test). Remarkably, the antennaviruses have the highest CAI_HHE_ values for NP and L, while the hartmaniviruses have the lowest values (Figure 6).

**Figure 7:**
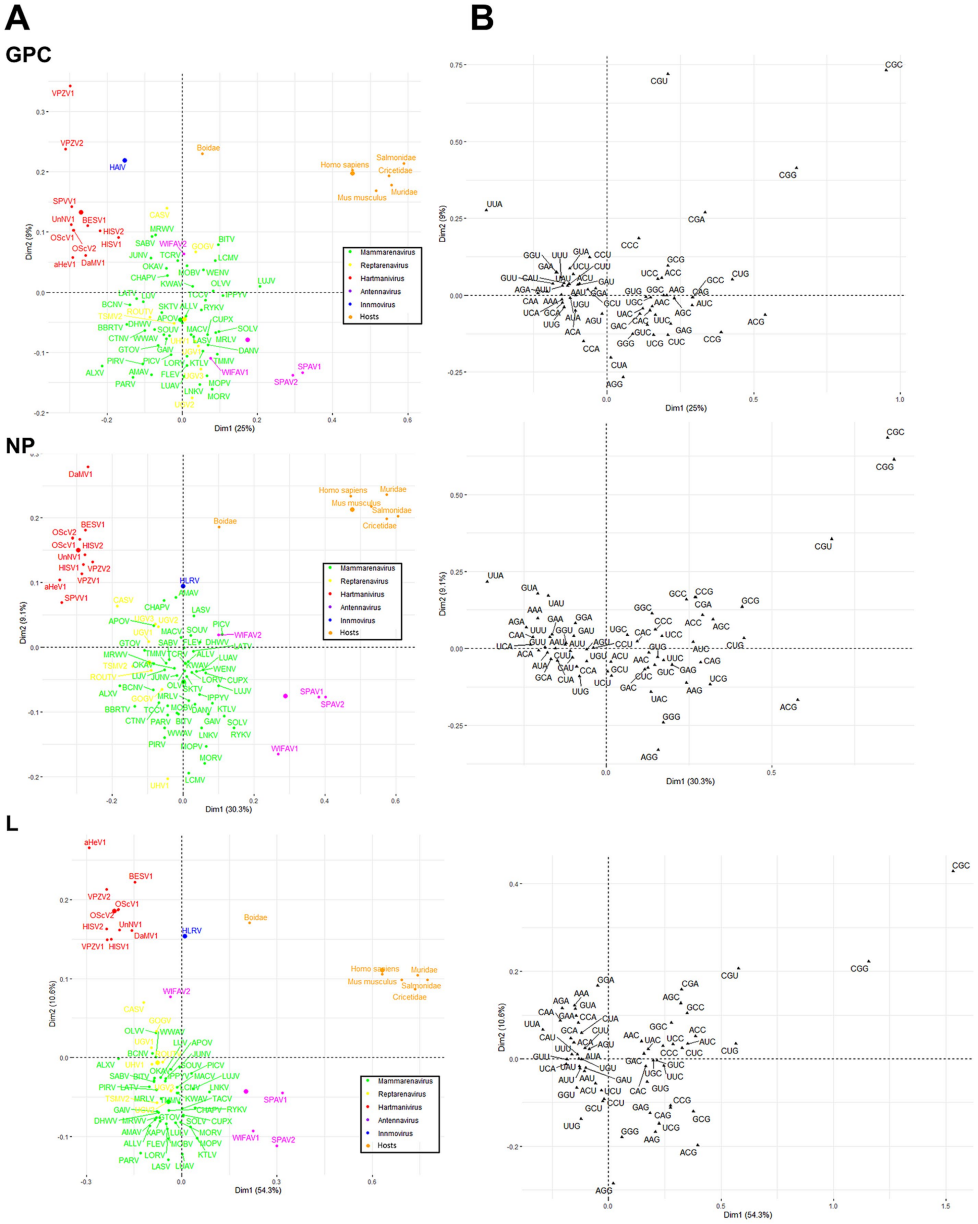
Correspondence analysis of GPC, NP and L protein sequences of arenavirus based on RSCU values. **(A)** Distribution of the genes of all arenaviruses on the plane corresponding to the coordinates of the first and second major axes. The colors correspond to the different genera as shown. The small dots correspond to the individual species, the large dots to the average per genus. **(B)** The distribution of synonymous codons is shown along the first and second axis of the correspondence analysis.

Finally, a correspondence analysis (CA) of the RSCU with the three viral proteins of each virus species and genus was performed to analyze the CUB patterns in more detail. The results showed that *Antennavirus* members exhibited a broader distribution, while *Hartmanivirus* showed a higher bias. All host proteins were located in the same region of the CA plot, with *Boidae* being further away from the other hosts and closer to the *Hartmanivirus* members. Mammarenaviruses and reptarenaviruses appear to have a similar codon usage bias as they are located close to each other. No clear correlation was found between the codon usage bias of the pathogenic species and the human CUB (Figure 7A). In particular, UUA (L), ACG (T), CGU (R), CGG (R) and CGC (R) were important in defining the CA axes for all arenavirus proteins (Figure 7B). UUA was significantly different for all genera, with *Hartmanivirus* having the highest and *Antennavirus* the lowest. In contrast, ACG was the preferred codon for S in *Antennavirus*. The dimensions in the CA indicate the different sources of variation between a set of multivariate data points. In this case, dimension 1 (Dim1) was different among proteins and explained almost 50% of the variation in L, while NP and GPC had lower values, while dimension 2 (Dim2) explained at most 10% of the variation in all proteins. It is interesting to note that the central axes (0, 0) divide the codons into two clusters: codons with either A or T at the third base position (AT3) on the left and codons with either G or C at the third base position (GC3) on the right (Figure 7B).

## Discussion

Our report provides a comprehensive analysis of codon usage bias for all arenavirus genera. By using a concatenated alignment of the L and NP proteins, we have constructed a phylogenetic tree that determines the distance in clustering between specific species within the family with greater precision than trees constructed using NP or L separately. As reported by Forni et al. (2018), our tree also places NW Arenavirus closer to the common ancestor of *Mammarenavirus*, and both OW and NW mammarenaviruses are in the same clade as *Reptarenavirus*, with the common ancestors of *Hartmanivirus* and *Antennavirus* being the most distant in the phylogeny. The fact that mammarenaviruses and reptarenaviruses are closer to each other in phylogeny is also reflected in their similarity in genome composition. Based on phylogeographic analyses, the possibility that OW and NW mammarenaviruses descended from a common ancestor that occurred in both Africa and South America has been proposed, with a reptilian arenavirus, such as reptarenaviruses, being an attractive candidate (Forni et al., 2018). Since the full range of natural hosts of mammarenaviruses and reptarenaviruses is unknown, another possibility is that their common ancestor infected a different mammalian host and that snakes are not the reservoir of the reptarenavirus, but an accidental host. This is supported by the analogy with the mammarenavirus, which can infect animal species other than natural hosts, at least in experimental settings, often leading to severe disease and even death (Forni et al., 2018; Pontremoli et al., 2019).

The total nucleotide content of the genome can strongly influence codon usage patterns (Jenkins and Holmes, 2003). Furthermore, van Hemert et al. (2016) suggested that nucleotide bias in RNA virus genomes is the primary determinant of specific codon usage, limiting the role of codon selection and translational control. Our analysis of the nucleotide composition of *GPC*, *NP* and *L* genes revealed that most codons containing the dinucleotide CG are highly repressed and those containing the dinucleotide UA are slightly underrepresented, with this difference being smaller in antennaviruses. This was to be expected as it has been reported for most RNA viruses (Karlin et al., 1994). CpG dinucleotides have been largely removed from the human genome (Cooper and Gerber-Huber, 1985), so that human mRNAs are CpG-repressed, a fact that is largely reflected by CpG suppression in viruses that infect humans, even though their genome composition may not have been shaped by DNA methylation/deamination (Cooper and Gerber-Huber, 1985). The selection pressure that has led to CpG suppression in viral genomes remains unknown (Goncalves-Carneiro et al., 2021). However, there are some reasons that could explain this, at least in part. In vertebrates, there are short, interspersed DNA sequences that deviate significantly from the average genomic pattern because they are GC- and CpG-rich and predominantly unmethylated (Deaton and Bird, 2011). These regions, known as CpG islands (CGI), are often sites of transcription initiation. For promoter function, they destabilize nucleosomes and attract proteins (Angeloni and Bogdanovic, 2021). In addition, CGIs have recently been linked to a gene regulatory mechanism in which CGIs are bound by a protein complex that specifically protects genic transcripts from premature termination, effectively distinguishing between genic and extragenic transcription and allowing normal gene expression (Hughes et al., 2023). It could be hypothesized that viral RNA with a low CG content interacts less with host proteins and is therefore more efficient for viral replication and translation. On the other hand, the antiviral host factor zinc finger protein (ZAP) has been reported to selectively inhibit replication of a variety of viruses by recognizing the CG-rich RNA sequences and activating the viral RNA degradation machinery (Pal et al., 2023). *Zap* and its analog *PARP12* genes arose early in vertebrate divergence and originated in an original gene whose progeny are present in some modern invertebrates such as cnidarians but absent in others such as arthropods (Goncalves-Carneiro et al., 2021), suggesting that it may be a mutational pressure in all known arenaviruses. It is hoped that future studies will clarify the role of CpG suppression in viral genomes.

A weak codon usage bias was observed in all *Arenaviridae* genera (ENC ~50). Although *Hartmanivirus* had lower ENC values, the corresponding points in the ENC plot were closer to the curve (predicted ENC value in the absence of natural selection), suggesting a greater effect of compositional and mutational biases compared to the other arenaviruses. The ENC analysis also suggests a more important role of selection in the case of *Mammarenavirus* and *Antennavirus*, as also shown by the corresponding neutrality plots. Although a weak codon usage bias has already been reported for the mammarenaviruses CHPV and SABV, it was concluded that mutational pressure has the strongest influence on codon usage bias in both hemorrhagic fever pathogens (Malhotra and Kumar, 2021). The difference between our results could be due to the fact that we created the neutrality plots for each protein and grouped the arenaviruses by genus to obtain more information, as we felt that only three proteins were too few to derive a good regression.

The role of mutation and selection was not the same for each protein in all genera. Our results suggest that although selection pressure remains the most important determinant of codon usage bias, it has a stronger influence on the codon usage bias of GPC and NP than on that of L in mammarenaviruses. The L protein has been described as the main driver of evolution of the genus as it is subject to positive selection at multiple sites and there is evidence that changes in its sequence lead to differential replication efficiency and disease phenotypes in rodents (Pontremoli et al., 2017; Forni et al., 2018), and, in the case of LASV, affect transmissibility or severity of the disease in humans (Pontremoli et al., 2019). It has also been hypothesized that selection pressure acting on arenavirus L proteins confers species-specific increased replication capacity or faster spread kinetics to the virus, facilitating escape from immune surveillance and allowing persistent infection (Sullivan et al., 2015). Interestingly, our results suggest that antennaviruses and reptarenaviruses do not follow this pattern and natural selection plays a relatively more important role than mutational pressure for the L protein.

Our CAI analysis showed good agreement with the general use of host codons, with humans and *Boidae* scoring higher for most viruses. In particular, hartmaniviruses and antennaviruses showed the highest CAI for all proteins when the proteins of their respective hosts were used as reference sets. *Hartmanivirus* is also positioned differently in the correspondence analysis as it is closer to the *Boidae*. This suggests an adaptation to host-specific codons, but does not explain the difference to *Reptarenavirus*, which has the same hosts, which requires further investigation. Remarkably, *Mammarenavirus* and *Reptarenavirus* had very similar nucleotide composition, codon usage bias and CAI values, both of which were markedly different from *Hartmanivirus*, which had the highest CAI within the *Boidae* for all its proteins. This reinforces the hypothesis that reptarenaviruses may have an unknown mammalian reservoir. In addition, mammalian cells have been successfully infected with reptarenaviruses (Hepojoki et al., 2015a), and recent data also suggest successful infection in the mouse model (Abba et al., 2017). Furthermore, for mammarenaviruses, there is limited evidence of host-virus co-divergence (Forni et al., 2018), suggesting that the shared host of *Reptarenavirus* and *Hartmanivirus* does not reflect a closer evolutionary link and providing another suitable explanation for the difference in codon usage behavior between these two genera.

The CAI was also calculated using a set of highly expressed human proteins as a reference. Since CAI correlates with protein expression (Sharp and Li, 1987), our results suggest that the arenavirus proteins analyzed have the potential for efficient translation in humans, at least in terms of optimizing codon usage. However, since we found no significant difference between CAI_HHE_ and CAI_HLE_ proteins, further studies should be performed to evaluate the expression levels of arenavirus proteins in human hosts.

In hantaviruses, it has been suggested that less adaptation to their hosts allows controlled and sustained infection in their natural reservoirs (Meyer and Schmaljohn, 2000). Such differences between hosts and natural reservoirs could correlate with the severe disease that certain mammarenaviruses cause when infecting humans, as opposed to the milder symptoms of infection in their reservoirs. In support of this hypothesis, we found that arenaviruses generally have lower CAI and CAI_HHE_ in *Muridae* and *Cricetidae* than in humans. In addition, codon deoptimization of LCMV GPC and NP was found to produce highly attenuated viruses but still provide protection against wild-type LCMV (Cheng et al., 2015; Cheng et al., 2017). However, disease severity of LASV could not be correlated with significantly different nCAI levels, suggesting, at least for LASV, that disease severity is mainly determined either by interindividual human variability or by viral factors other than nCAI (Pontremoli et al., 2017). Future studies need to consider other factors influencing codon usage bias, as has been hypothesized for other viruses, e.g., geographic location and gene function (Chen et al., 2014; Rahman et al., 2018).

Finally, our correspondence analysis (CA) of individual RSCU codons reveals two clusters similar to those described in human mRNA that are rich in GC3 and AT3 and are associated with stabilization or destabilization of mRNA through interaction with interleukin enhancer binding factor 2 (ILF2) and perhaps ILF3 (Hia et al., 2019). The extent to which these mechanisms are effective in arenaviral RNA infections must be clarified in the future.

## Conclusion

Here we report the codon usage pattern of the *Arenaviridae* family common proteins as a whole. We found a weak codon usage bias, as has been reported for other RNA viruses, with ENC and neutrality diagrams indicating an important role of selection in evolution. As with all arenaviruses (and most small eukaryotic viruses), strong repression of CG-containing codons was observed. *Antennavirus* and *Hartmanivirus* showed different behavior within the family, both in composition and codon usage pattern. Remarkably, all arenaviruses appear to have codon usage adapted to both *H. sapiens* and *Boidae*, and some degree of host adaptation of codon usage patterns was observed for *Hartmanivirus* and *Antennavirus*.

## Data Availability

The datasets presented in this study can be found in online repositories. The names of the repository/repositories and accession number(s) can be found in the article/Supplementary material.

## Footnotes

^1^ http://www.ncbi.nlm.nih.gov/GenBank

^2^ https://www.proteinatlas.org

^3^ https://ftp.ensembl.org/pub/release111/fasta/homosapiens/cds/Homosapiens.GRCh38.cds.all.fa.gz

^4^ https://gtexportal.org/

^5^ http://genomes.urv.es/CAIcal/

^6^ www.graphpad.com

## References

[ref1] Abba Y. Hassim H. Hamzah H. Ibrahim O. E. Mohd Lila M. A. Noordin M. M. (2017). Pathological vicissitudes and oxidative stress enzyme responses in mice experimentally infected with reptarenavirus (isolate UPM/MY01). Microb. Pathog. 104, 17–27. doi: 10.1016/j.micpath.2017.01.003, PMID: 28062291

[ref2] Angeloni A. Bogdanovic O. (2021). Sequence determinants, function, and evolution of CpG islands. Biochem. Soc. Trans. 49, 1109–1119. doi: 10.1042/BST20200695, PMID: 34156435 PMC8286816

[ref3] Baha S. Behloul N. Liu Z. Wei W. Shi R. Meng J. (2019). Comprehensive analysis of genetic and evolutionary features of the hepatitis E virus. BMC Genomics 20:790. doi: 10.1186/s12864-019-6100-8, PMID: 31664890 PMC6820953

[ref4] Belalov I. S. Lukashev A. N. (2013). Causes and implications of codon usage bias in RNA viruses. PLoS One 8:e56642. doi: 10.1371/journal.pone.0056642, PMID: 23451064 PMC3581513

[ref5] Bulmer M. (1991). The selection-mutation-drift theory of synonymous codon usage. Genetics 129, 897–907. doi: 10.1093/genetics/129.3.897, PMID: 1752426 PMC1204756

[ref6] Butt A. M. Nasrullah I. Qamar R. Tong Y. (2016). Evolution of codon usage in Zika virus genomes is host and vector specific. Emerg. Microbes Infect. 5:e107. doi: 10.1038/emi.2016.106, PMID: 27729643 PMC5117728

[ref7] Chaney J. L. Clark P. L. (2015). Roles for synonymous codon usage in protein biogenesis. Annu. Rev. Biophys. 44, 143–166. doi: 10.1146/annurev-biophys-060414-034333, PMID: 25747594

[ref8] Chen Y. M. Hu S. J. Lin X. D. Tian J. H. Lv J. X. Wang M. R.. (2023). Host traits shape virome composition and virus transmission in wild small mammals. Cell 186, 4662–4675.e12. doi: 10.1016/j.cell.2023.08.029, PMID: 37734372

[ref9] Chen Y. M. Sadiq S. Tian J. H. Chen X. Lin X. D. Shen J. J.. (2022). RNA viromes from terrestrial sites across China expand environmental viral diversity. Nat. Microbiol. 7, 1312–1323. doi: 10.1038/s41564-022-01180-2, PMID: 35902778

[ref10] Chen Y. Shi Y. Deng H. Gu T. Xu J. Ou J.. (2014). Characterization of the porcine epidemic diarrhea virus codon usage bias. Infect. Genet. Evol. 28, 95–100. doi: 10.1016/j.meegid.2014.09.004, PMID: 25239728 PMC7185563

[ref11] Cheng B. Y. H. Nogales A. de la Torre J. C. Martinez-Sobrido L. (2017). Development of live-attenuated arenavirus vaccines based on codon deoptimization of the viral glycoprotein. Virology 501, 35–46. doi: 10.1016/j.virol.2016.11.001, PMID: 27855284 PMC5201438

[ref12] Cheng B. Y. Ortiz-Riano E. Nogales A. de la Torre J. C. Martinez-Sobrido L. (2015). Development of live-attenuated arenavirus vaccines based on codon deoptimization. J. Virol. 89, 3523–3533. doi: 10.1128/JVI.03401-14, PMID: 25589652 PMC4403387

[ref13] Christensen K. A. Rondeau E. B. Minkley D. R. Sakhrani D. Biagi C. A. Flores A. M.. (2020). The sockeye salmon genome, transcriptome, and analyses identifying population defining regions of the genome. PLoS One 15:e0240935. doi: 10.1371/journal.pone.0240935, PMID: 33119641 PMC7595290

[ref14] Cooper D. N. Gerber-Huber S. (1985). DNA methylation and CpG suppression. Cell Differ. 17, 199–205. doi: 10.1016/0045-6039(85)90488-9, PMID: 3902251

[ref15] Deaton A. M. Bird A. (2011). CpG islands and the regulation of transcription. Genes Dev. 25, 1010–1022. doi: 10.1101/gad.2037511, PMID: 21576262 PMC3093116

[ref16] dos Reis M. Savva R. Wernisch L. (2004). Solving the riddle of codon usage preferences: a test for translational selection. Nucleic Acids Res. 32, 5036–5044. doi: 10.1093/nar/gkh834, PMID: 15448185 PMC521650

[ref17] Dutta R. Buragohain L. Borah P. (2020). Analysis of codon usage of severe acute respiratory syndrome corona virus 2 (SARS-CoV-2) and its adaptability in dog. Virus Res. 288:198113. doi: 10.1016/j.virusres.2020.198113, PMID: 32771430 PMC7410794

[ref18] Elek A. Kuzman M. Vlahoviček K. (2018). "coRdon: codon usage analysis and prediction of gene expressivity". Available at: https://github.com/BioinfoHR/coRdon (Accessed February 21, 2024).

[ref19] Emonet S. Lemasson J. J. Gonzalez J. P. de Lamballerie X. Charrel R. N. (2006). Phylogeny and evolution of old world arenaviruses. Virology 350, 251–257. doi: 10.1016/j.virol.2006.01.026 16494913

[ref20] Forni D. Pontremoli C. Pozzoli U. Clerici M. Cagliani R. Sironi M. (2018). Ancient evolution of Mammarenaviruses: adaptation via changes in the L protein and no evidence for host-virus Codivergence. Genome Biol. Evol. 10, 863–874. doi: 10.1093/gbe/evy050, PMID: 29608723 PMC5863214

[ref21] Goncalves-Carneiro D. Takata M. A. Ong H. Shilton A. Bieniasz P. D. (2021). Origin and evolution of the zinc finger antiviral protein. PLoS Pathog. 17:e1009545. doi: 10.1371/journal.ppat.1009545, PMID: 33901262 PMC8102003

[ref22] Gonzalez J. P. Emonet S. de Lamballerie X. Charrel R. (2007). Arenaviruses. Curr. Top. Microbiol. Immunol. 315, 253–288. doi: 10.1007/978-3-540-70962-6_11, PMID: 17848068 PMC7122678

[ref23] Grismer J. L. Escalona M. Miller C. Beraut E. Fairbairn C. W. Marimuthu M. P. A.. (2022). Reference genome of the rubber boa, *Charina bottae* (Serpentes: Boidae). J. Hered. 113, 641–648. doi: 10.1093/jhered/esac048, PMID: 36056886 PMC9709994

[ref24] Harrison P. W. Amode M. R. Austine-Orimoloye O. Azov A. G. Barba M. Barnes I.. (2024). Ensembl 2024. Nucleic Acids Res. 52, D891–D899. doi: 10.1093/nar/gkad1049, PMID: 37953337 PMC10767893

[ref25] He M. An T. Z. Teng C. B. (2014). Evolution of mammalian and avian bornaviruses. Mol. Phylogenet. Evol. 79, 385–391. doi: 10.1016/j.ympev.2014.07.006, PMID: 25046276

[ref26] Hepojoki J. Kipar A. Korzyukov Y. Bell-Sakyi L. Vapalahti O. Hetzel U. (2015a). Replication of boid inclusion body disease-associated arenaviruses is temperature sensitive in both boid and mammalian cells. J. Virol. 89, 1119–1128. doi: 10.1128/JVI.03119-14, PMID: 25378485 PMC4300630

[ref27] Hepojoki J. Salmenpera P. Sironen T. Hetzel U. Korzyukov Y. Kipar A.. (2015b). Arenavirus coinfections are common in snakes with Boid inclusion body disease. J. Virol. 89, 8657–8660. doi: 10.1128/JVI.01112-15, PMID: 26041290 PMC4524219

[ref28] Hetzel U. Sironen T. Laurinmaki P. Liljeroos L. Patjas A. Henttonen H.. (2013). Isolation, identification, and characterization of novel arenaviruses, the etiological agents of boid inclusion body disease. J. Virol. 87, 10918–10935. doi: 10.1128/JVI.01123-13, PMID: 23926354 PMC3807292

[ref29] Hia F. Yang S. F. Shichino Y. Yoshinaga M. Murakawa Y. Vandenbon A.. (2019). Codon bias confers stability to human mRNAs. EMBO Rep. 20:e48220. doi: 10.15252/embr.201948220, PMID: 31482640 PMC6831995

[ref30] Hughes A. L. Szczurek A. T. Kelley J. R. Lastuvkova A. Turberfield A. H. Dimitrova E.. (2023). A CpG island-encoded mechanism protects genes from premature transcription termination. Nat. Commun. 14:726. doi: 10.1038/s41467-023-36236-2, PMID: 36759609 PMC9911701

[ref31] Husson F. Josse J. Lê S. d'Agrocampus R. Mazet J. (2008). FactoMineR: an R package for multivariate analysis. J. Stat. Soft. 25, 1–18. doi: 10.18637/jss.v025.i01

[ref32] Ikemura T. (1981). Correlation between the abundance of *Escherichia coli* transfer RNAs and the occurrence of the respective codons in its protein genes. J. Mol. Biol. 146, 1–21. doi: 10.1016/0022-2836(81)90363-6, PMID: 6167728

[ref33] Jenkins G. M. Holmes E. C. (2003). The extent of codon usage bias in human RNA viruses and its evolutionary origin. Virus Res. 92, 1–7. doi: 10.1016/s0168-1702(02)00309-x 12606071

[ref34] Jenkins G. M. Pagel M. Gould E. A. de Zanotto A. P. M. Holmes E. C. (2001). Evolution of base composition and codon usage bias in the genus Flavivirus. J. Mol. Evol. 52, 383–390. doi: 10.1007/s002390010168, PMID: 11343134

[ref35] Karlin S. Doerfler W. Cardon L. R. (1994). Why is CpG suppressed in the genomes of virtually all small eukaryotic viruses but not in those of large eukaryotic viruses? J. Virol. 68, 2889–2897. doi: 10.1128/JVI.68.5.2889-2897.1994, PMID: 8151759 PMC236777

[ref36] Kassambara A. Mundt F. (2020). Factoextra: extract and visualize the results of multivariate data analyses. Available at: https://CRAN.R-project.org/package=factoextra (Accessed January 23, 2024).

[ref37] Katoh K. Rozewicki J. Yamada K. D. (2019). MAFFT online service: multiple sequence alignment, interactive sequence choice and visualization. Brief. Bioinform. 20, 1160–1166. doi: 10.1093/bib/bbx108, PMID: 28968734 PMC6781576

[ref38] Khandia R. Singhal S. Kumar U. Ansari A. Tiwari R. Dhama K.. (2019). Analysis of Nipah virus codon usage and adaptation to hosts. Front. Microbiol. 10:886. doi: 10.3389/fmicb.2019.00886, PMID: 31156564 PMC6530375

[ref39] Luo W. Roy A. Guo F. Irwin D. M. Shen X. Pan J.. (2020). Host adaptation and evolutionary analysis of Zaire ebolavirus: insights from codon usage based investigations. Front. Microbiol. 11:570131. doi: 10.3389/fmicb.2020.570131, PMID: 33224111 PMC7674656

[ref40] Luo W. Tian L. Huang C. Li J. Shen X. Murphy R. W.. (2019). The codon usage bias of avian influenza A viruses. J. Infect. 79, 174–187. doi: 10.1016/j.jinf.2019.05.003, PMID: 31075292

[ref41] Malhotra H. Kumar A. (2021). Codon usage signatures in Sabia and Chapare for host adaptation. Bioinformation 17, 891–898. doi: 10.6026/97320630017891 35574503 PMC9070627

[ref42] Meyer B. J. Schmaljohn C. S. (2000). Persistent Hantavirus infections: characteristics and mechanisms. Trends Microbiol. 8, 61–67. doi: 10.1016/s0966-842x(99)01658-3, PMID: 10664598

[ref43] Mogro E. G. Bottero D. Lozano M. J. (2022). Analysis of SARS-CoV-2 synonymous codon usage evolution throughout the COVID-19 pandemic. Virology 568, 56–71. doi: 10.1016/j.virol.2022.01.011, PMID: 35134624 PMC8808327

[ref44] Mordecai G. J. Miller K. M. Di Cicco E. Schulze A. D. Kaukinen K. H. Ming T. J.. (2019). Endangered wild salmon infected by newly discovered viruses. eLife 8:615. doi: 10.7554/eLife.47615, PMID: 31478480 PMC6721791

[ref45] Nasrullah I. Butt A. M. Tahir S. Idrees M. Tong Y. (2015). Genomic analysis of codon usage shows influence of mutation pressure, natural selection, and host features on Marburg virus evolution. BMC Evol. Biol. 15:174. doi: 10.1186/s12862-015-0456-4, PMID: 26306510 PMC4550055

[ref46] Nguyen T. H. Wang D. Rahman S. U. Bai H. Yao X. Chen D.. (2021). Analysis of codon usage patterns and influencing factors in rice tungro bacilliform virus. Infect. Genet. Evol. 90:104750. doi: 10.1016/j.meegid.2021.104750, PMID: 33548490

[ref47] Pal S. Kumar A. Vashisth H. (2023). Role of dynamics and mutations in interactions of a zinc finger antiviral protein with CG-rich viral RNA. J. Chem. Inf. Model. 63, 1002–1011. doi: 10.1021/acs.jcim.2c01487, PMID: 36707411 PMC10129844

[ref48] Plotkin J. B. Kudla G. (2011). Synonymous but not the same: the causes and consequences of codon bias. Nat. Rev. Genet. 12, 32–42. doi: 10.1038/nrg2899, PMID: 21102527 PMC3074964

[ref49] Pontremoli C. Forni D. Cagliani R. Pozzoli U. Riva S. Bravo I. G.. (2017). Evolutionary analysis of Old World arenaviruses reveals a major adaptive contribution of the viral polymerase. Mol. Ecol. 26, 5173–5188. doi: 10.1111/mec.14282, PMID: 28779541

[ref50] Pontremoli C. Forni D. Sironi M. (2019). Arenavirus genomics: novel insights into viral diversity, origin, and evolution. Curr. Opin. Virol. 34, 18–28. doi: 10.1016/j.coviro.2018.11.001, PMID: 30497052

[ref51] Puigbo P. Bravo I. G. Garcia-Vallve S. (2008). CAIcal: a combined set of tools to assess codon usage adaptation. Biol. Direct 3:38. doi: 10.1186/1745-6150-3-38, PMID: 18796141 PMC2553769

[ref52] Radoshitzky S. R. Buchmeier M. J. Charrel R. N. Clegg J. C. S. Gonzalez J. J. Gunther S.. (2019). ICTV Virus Taxonomy Profile: Arenaviridae. J. Gen. Virol. 100, 1200–1201. doi: 10.1099/jgv.0.001280, PMID: 31192784 PMC12139605

[ref53] Radoshitzky S. R. Buchmeier M. J. Charrel R. N. Gonzalez J. J. Gunther S. Hepojoki J.. (2023). ICTV Virus Taxonomy Profile: Arenaviridae 2023. J. Gen. Virol. 104:891. doi: 10.1099/jgv.0.001891, PMID: 37698490 PMC10720992

[ref54] Rahman S. U. Yao X. Li X. Chen D. Tao S. (2018). Analysis of codon usage bias of Crimean-Congo hemorrhagic fever virus and its adaptation to hosts. Infect. Genet. Evol. 58, 1–16. doi: 10.1016/j.meegid.2017.11.027, PMID: 29198972

[ref55] RoyChoudhury S. Mukherjee D. (2013). Complex codon usage pattern and compositional features of retroviruses. Comput. Math. Methods Med. 2013:848123. doi: 10.1155/2013/848123, PMID: 24288576 PMC3833384

[ref56] RStudioTeam (2020). RStudio: Integrated development environment for R. Boston, MA: RStudio.

[ref57] Salazar-Bravo J. Ruedas L. A. Yates T. L. (2002). Mammalian reservoirs of arenaviruses. Curr. Top. Microbiol. Immunol. 262, 25–63. doi: 10.1007/978-3-642-56029-3_2, PMID: 11987807

[ref58] Sarute N. Ross S. R. (2017). New World arenavirus biology. Annu. Rev. Virol. 4, 141–158. doi: 10.1146/annurev-virology-101416-042001, PMID: 28645238 PMC7478856

[ref59] Sharp P. M. Li W. H. (1987). The codon adaptation index--a measure of directional synonymous codon usage bias, and its potential applications. Nucleic Acids Res. 15, 1281–1295. doi: 10.1093/nar/15.3.1281, PMID: 3547335 PMC340524

[ref60] Shi M. Lin X. D. Chen X. Tian J. H. Chen L. J. Li K.. (2018). The evolutionary history of vertebrate RNA viruses. Nature 556, 197–202. doi: 10.1038/s41586-018-0012-7, PMID: 29618816

[ref61] Stenglein M. D. Sanders C. Kistler A. L. Ruby J. G. Franco J. Y. Reavill D. R.. (2012). Identification, characterization, and in vitro culture of highly divergent arenaviruses from boa constrictors and annulated tree boas: candidate etiological agents for snake inclusion body disease. MBio 3, e00180–e00112. doi: 10.1128/mBio.00180-12, PMID: 22893382 PMC3419519

[ref62] Sullivan B. M. Teijaro J. R. de la Torre J. C. Oldstone M. B. (2015). Early virus-host interactions dictate the course of a persistent infection. PLoS Pathog. 11:e1004588. doi: 10.1371/journal.ppat.1004588, PMID: 25569216 PMC4287607

[ref63] Tort F. L. Castells M. Cristina J. (2020). A comprehensive analysis of genome composition and codon usage patterns of emerging coronaviruses. Virus Res. 283:197976. doi: 10.1016/j.virusres.2020.197976, PMID: 32294518 PMC7152894

[ref64] Trifinopoulos J. Nguyen L. T. von Haeseler A. Minh B. Q. (2016). W-IQ-TREE: a fast online phylogenetic tool for maximum likelihood analysis. Nucleic Acids Res. 44, W232–W235. doi: 10.1093/nar/gkw256, PMID: 27084950 PMC4987875

[ref65] Uhlen M. Fagerberg L. Hallstrom B. M. Lindskog C. Oksvold P. Mardinoglu A.. (2015). Proteomics. Tissue-based map of the human proteome. Science 347:1260419. doi: 10.1126/science.1260419, PMID: 25613900

[ref66] van Hemert F. van der Kuyl A. C. Berkhout B. (2016). Impact of the biased nucleotide composition of viral RNA genomes on RNA structure and codon usage. J. Gen. Virol. 97, 2608–2619. doi: 10.1099/jgv.0.000579, PMID: 27519195

[ref67] Velazquez-Salinas L. Zarate S. Eschbaumer M. Pereira Lobo F. Gladue D. P. Arzt J.. (2016). Selective factors associated with the evolution of codon usage in natural populations of arboviruses. PLoS One 11:e0159943. doi: 10.1371/journal.pone.0159943, PMID: 27455096 PMC4959722

[ref68] Wang H. Liu S. Zhang B. Wei W. (2016). Analysis of synonymous codon usage Bias of Zika virus and its adaption to the hosts. PLoS One 11:e0166260. doi: 10.1371/journal.pone.0166260, PMID: 27893824 PMC5125587

[ref69] Wright F. (1990). The 'effective number of codons' used in a gene. Gene 87, 23–29. doi: 10.1016/0378-1119(90)90491-9, PMID: 2110097

[ref70] Zhang X. Cai Y. Zhai X. Liu J. Zhao W. Ji S.. (2018). Comprehensive analysis of codon usage on rabies virus and other lyssaviruses. Int. J. Mol. Sci. 19:2397. doi: 10.3390/ijms19082397, PMID: 30110957 PMC6121662

[ref71] Zhang L. Kasif S. Cantor C. R. Broude N. E. (2004). GC/AT-content spikes as genomic punctuation marks. Proc. Natl. Acad. Sci. USA 101, 16855–16860. doi: 10.1073/pnas.0407821101, PMID: 15548610 PMC534751

[ref72] Zhang J. Wang M. Liu W. Q. Zhou J. H. Chen H. T. Ma L. N.. (2011). Analysis of codon usage and nucleotide composition bias in polioviruses. Virol. J. 8:146. doi: 10.1186/1743-422X-8-146, PMID: 21450075 PMC3079669

