# Supplementary material for: A study on the codon usage bias of arenavirus common genes
Source: Front Microbiol. 2025 Jan 23;15:1490076. doi: 10.3389/fmicb.2024.1490076 (PMC11799557; doi:10.3389/fmicb.2024.1490076)
Supplement: Supplementary file 1 [file Data_Sheet_1.pdf]

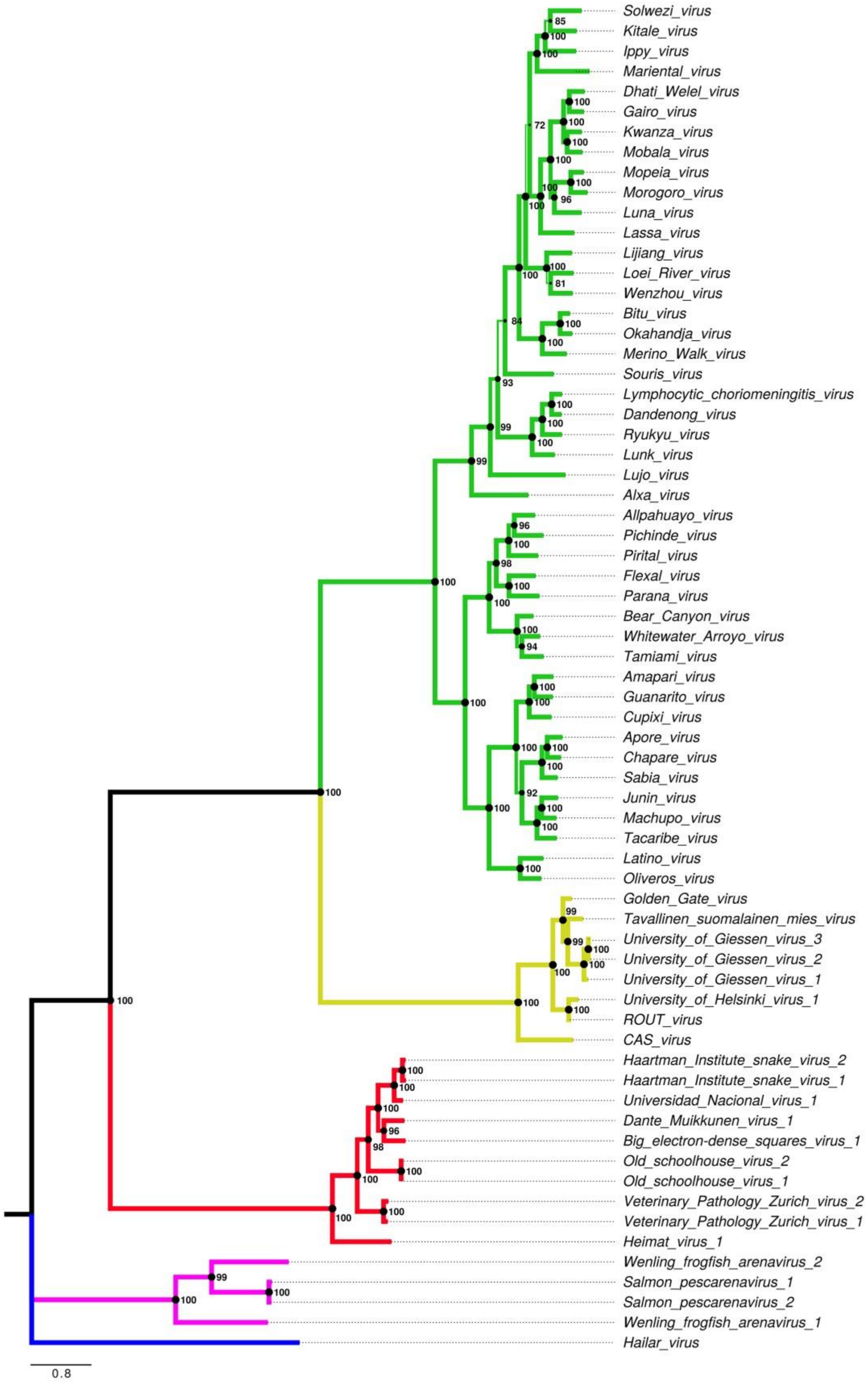
Solwezi_virus
Kitale_virus
Ippy_virus
Mariental_virus
Dhati_Welel_virus
Gairo_virus
Kwanza_virus
Mobala_virus
Mopeia_virus
Morogoro_virus
Luna_virus
Lassa_virus
Lijiang_virus
Loei_River_virus
Wenzhou_virus
Bitu_virus
Okahandja_virus
Merino_Walk_virus
Souris_virus
Lymphocytic_choriomeningitis_virus
Dandenong_virus
Ryukyu_virus
Lunk_virus
Lujo_virus
Alxa_virus
Allpahuayo_virus
Pichinde_virus
Pirital_virus
Flexal_virus
Parana_virus
Bear_Canyon_virus
Whitewater_Arroyo_virus
Tamiami_virus
Amapari_virus
Guanarito_virus
Cupixi_virus
Apore_virus
Chapare_virus
Sabia_virus
Junin_virus
Machupo_virus
Tacaribe_virus
Latino_virus
Oliveros_virus
Golden_Gate_virus
Tavallinen_suomalainen_mies_virus
University_of_Giessen_virus_3
University_of_Giessen_virus_2
University_of_Giessen_virus_1
University_of_Helsinki_virus_1
ROUT_virus
CAS_virus
Haartman_Institute_snake_virus_2
Haartman_Institute_snake_virus_1
Universidad_Nacional_virus_1
Dante_Muikkunen_virus_1
Big_electron-dense_squares_virus_1
Old_schoolhouse_virus_2
Old_schoolhouse_virus_1
Veterinary_Pathology_Zurich_virus_2
Veterinary_Pathology_Zurich_virus_1
Heimat_virus_1
Wenling_frogfish_arenavirus_2
Salmon_pescarenavirus_1
Salmon_pescarenavirus_2
Wenling_frogfish_arenavirus_1
Hailar_virus
0.8

Figure S1. Maximum likelihood tree of arenavirus. The tree was constructed from the concatenated L and NP protein sequences of the arenaviruses recognized and accepted by the ICTV. The genomic sequences were downloaded from the NCBI virus database, aligned using MAFFT and a maximum likelihood phylogenetic tree was created using IQTree. The genera are highlighted by different colors. Green: *Mammarenavirus*. Yellow: *Reptarenavirus*. Red: *Hartmanivirus*. Purple: *Antennavirus*. Blue: *Innmovirus*. The numbers indicate the bootstrap support for each node.
