# Supplementary material for: A study on the codon usage bias of arenavirus common genes
Source: Front Microbiol. 2025 Jan 23;15:1490076. doi: 10.3389/fmicb.2024.1490076 (PMC11799557; doi:10.3389/fmicb.2024.1490076)
Supplement: Supplementary file 2 [file Data_Sheet_2.pdf]

Supplementary Table 1. Virus genus and names and their abbreviations.

Genus	Species	Organism name	Abbrev.
Antennavirus	*Antennavirus salmonis*	Salmon pescarenavirus 1	SPAV1
Antennavirus	*Antennavirus salmonis*	Salmon pescarenavirus 2	SPAV2
Antennavirus	*Antennavirus hirsutum*	Wēnlǐng frogfish arenavirus 2	WlFAV2
Antennavirus	*Antennavirus striale*	Wēnlǐng frogfish arenavirus 1	WlFAV1
Hartmanivirus	*Hartmanivirus patriae*	andere Heimat virus 1	aHeV1
Hartmanivirus	*Hartmanivirus turici*	Veterinary pathology Zurich virus	VPZV1
Hartmanivirus	*Hartmanivirus turici*	Veterinary pathology Zurich virus	VPZV2
Hartmanivirus	*Hartmanivirus quadrati*	big electron-dense squares virus	BESV1
Hartmanivirus	*Hartmanivirus helvetiae*	Dante Muikkunen virus 1	DaMV1
Hartmanivirus	*Hartmanivirus scholae*	Old schoolhouse virus 1	OScV1
Hartmanivirus	*Hartmanivirus scholae*	Old schoolhouse virus 2	OScV2
Innmovirus	*Innmovirus hailarense*	Hailar virus	HLRV
Mammarenavirus	*Mammarenavirus*	Allpahuayo virus	ALLV
Mammarenavirus	*Mammarenavirus caliense*	Pichindé virus	PICHV
Mammarenavirus	*Mammarenavirus piritalense*	Pirital virus	PIRV
Mammarenavirus	*Mammarenavirus flexalense*	Flexal virus	FLEV
Mammarenavirus	*Mammarenavirus paranaense*	Paraná virus	PRAV
Mammarenavirus	*Mammarenavirus amapariense*	Amaparí virus	AMAV
Mammarenavirus	*Mammarenavirus cupixiense*	Cupixi virus	CUPXV
Mammarenavirus	*Mammarenavirus guanaritoense*	Guanarito virus	GTOV
Mammarenavirus	*Mammarenavirus aporeense*	Aporé virus	APOV
Mammarenavirus	*Mammarenavirus chapareense*	Chapare virus	CHAPV
Mammarenavirus	*Mammarenavirus brazilense*	Sabiá virus	SBAV
Mammarenavirus	*Mammarenavirus juninense*	Junín virus	JUNV
Mammarenavirus	*Mammarenavirus machupoense*	Machupo virus	MACV
Mammarenavirus	*Mammarenavirus tacaribeense*	Tacaribe virus	TCRV
Mammarenavirus	*Mammarenavirus latinum*	Latino virus	LATV
Mammarenavirus	*Mammarenavirus oliverosense*	Oliveros virus	OLVV
Mammarenavirus	*Mammarenavirus bearense*	Bear Canyon virus	BCNV
Mammarenavirus	*Mammarenavirus tamiamiense*	Tamiami virus	TMMV
Mammarenavirus	*Mammarenavirus*	Whitewater Arroyo virus	WWAV
Mammarenavirus	*Mammarenavirus alashanense*	Alxa virus	ALXV
Mammarenavirus	*Mammarenavirus bituense*	Bitu virus	BITV
Mammarenavirus	*Mammarenavirus merinoense*	Merino Walk virus	MRWV
Mammarenavirus	*Mammarenavirus okahandjaense*	Okahandja virus	OKAV
Mammarenavirus	*Dhati Welel mammarenavirus*	Dhati Welel virus	DHWV
Mammarenavirus	*Mammarenavirus gairoense*	Gairo virus	GAIV
Mammarenavirus	*Mammarenavirus kwanzaense*	Kwanza virus	KWAV
Mammarenavirus	*Mammarenavirus praomyidis*	Mobala virus	MOBV
Mammarenavirus	*Mammarenavirus lunaense*	Luna virus	LUAV
Mammarenavirus	*Mammarenavirus mopeiaense*	Mopeia virus	MOPV
Mammarenavirus	*Mammarenavirus mopeiaense*	Morogoro virus	MORV
Mammarenavirus	*Mammarenavirus lassaense*	Lassa virus	LASV
Mammarenavirus	*Mammarenavirus ippyense*	Ippy virus	IPPYV

Mammarenavirus	*Mammarenavirus kitaleense*	Kitale virus	KTLV
Mammarenavirus	*Mammarenavirus solweziense*	Solwezi virus	SOLV
Mammarenavirus	*Mammarenavirus marientalense*	Mariental virus	MRLV
Mammarenavirus.	*Mammarenavirus lijiangense*	Lìjiāng virus	LIJV
Mammarenavirus	*Mammarenavirus loeiense*	Loei River virus	LORV
Mammarenavirus	*Mammarenavirus wenzhouense*	Wēnzhōu virus	WENV
Mammarenavirus	*Mammarenavirus cameroonense*	Souris virus	SOUV
Mammarenavirus	*Mammarenavirus spp.*	Dandenong virus	DANV
Mammarenavirus	*Mammarenavirus lunkense*	Lunk virus	LNKV
Mammarenavirus	*Mammarenavirus*	Lymphocytic choriomeningitis	LCMV
Mammarenavirus.	*Mammarenavirus ryukyuense*	Ryukyu virus	RYKV
Mammarenavirus.	*Mammarenavirus lujoense*	Lujo virus	LUJV
Reptarenavirus	*Reptarenavirus rotterdamense*	ROUT virus	ROUTV
Reptarenavirus	*Alethinophid 2 reptarenavirus*	University of Helsinki virus	UHV1
Reptarenavirus	*Reptarenavirus commune*	Tavallinen suomalainen mies	TSMV2
Reptarenavirus	*Reptarenavirus giessenae*	University of Giessen virus 1	UGV1
Reptarenavirus	*Reptarenavirus giessenae*	University of Giessen virus 2	UGV2
Reptarenavirus	*Reptarenavirus giessenae*	University of Giessen virus 3	UGV3
Reptarenavirus	*Reptarenavirus aurei*	Golden Gate virus	GOGV
Reptarenavirus	*Reptarenavirus californiae*	CAS virus	CASV
Hartmanivirus	*Hartmanivirus haartmani*	Haartman Institute snake virus 1	HISV1
Hartmanivirus	*Haartman Institute snake virus 2*	Haartman Institute snake virus 2	HISV2
Hartmanivirus	*Hartmanivirus brazilense*	SetPatVet virus 1	SPVV1
Hartmanivirus	*Universidad Nacional virus 1*	Universidad Nacional virus 1	UnNV1
Mammarenavirus	*Mammarenavirus*	Big Brushy Tank virus	BBRTV
Mammarenavirus	*Mammarenavirus*	Catarina virus	CTNV
Mammarenavirus	*Mammarenavirus lunaense*	Luli virus	LULV
Mammarenavirus	*Mammarenavirus*	Pinhal virus	---
Mammarenavirus	*Mammarenavirus*	Skinner Tank virus	SKTV
Mammarenavirus	*Mammarenavirus*	Tonto Creek virus	TTCV
Mammarenavirus	*Mammarenavirus xapuriense*	Xapuri virus	XAPV
