# Supplementary material for: A study on the codon usage bias of arenavirus common genes
Source: Front Microbiol. 2025 Jan 23;15:1490076. doi: 10.3389/fmicb.2024.1490076 (PMC11799557; doi:10.3389/fmicb.2024.1490076)
Supplement: Supplementary file 3 [file Data_Sheet_3.PDF]

Supplementary Table 2. Virus hosts, host families, and accession numbers for viral genome segments used per virus (abbreviated name).

Virus	Host	Host Family	Accession numbers*
SPAV1	*Oncorhynchus tshawytscha*	*Salmonidae*	L: MK611982; M: MK611983; S: MK611984
SPAV2	*Oncorhynchus nerka*	*Salmonidae*	L: MK611981; M: MK611979; S: MK611980
WlFAV2	*Antennarius striatus*	*Antennariidae*	L: MG599866; M: MG599868; S: MG599867
WlFAV1	*Antennarius striatus*	*Antennariidae*	L: MG599863; M: MG599865; S: MG599864
aHeV1	*Boa constrictor*	*Boidae*	L: MN567062; S: MN567061
VPZV1	*Boa constrictor*	*Boidae*	L: MH483032; S: MH483031
VPZV2	*Boa constrictor*	*Boidae*	L: MH483044; S: MH483043
BESV1	*Boa constrictor*	*Boidae*	L: OM456564; S: OM456563
DaMV1	*Boa constrictor*	*Boidae*	L: MH778629; S: MH483026
OScV1	*Boa constrictor*	*Boidae*	L: MH483025; S: MH483024
OScV2	*Boa constrictor*	*Boidae*	L: MH483030; S: MH483029
HLRV	*river sediment*		L: MW896845; M: MW896846; S: MW896847
ALLV	*Oecomys bicolor*	*Cricetidae*	L: AY216502; S: AY012687
PICHV	*Nephelomys albigularis (Oryzomys albigularis)*	*Cricetidae*	L: AF427517; S: K02734
PIRV	*Sigmodon alstoni; Zygodontomys brevicauda*	*Cricetidae*	L: AY494081; S: AF485262
FLEV	*Hylaeamys megacephalus*	*Cricetidae*	L: EU627611; S: AF512831
PRAV	*Sooretamys angouya (Oryzomys)*	*Cricetidae*	L: EU627613; S: AF485261
AMAV	*Neacomys guianae*	*Cricetidae*	L: AY216517; S: AF485256
CUPXV	*Hylaeamys megacephalus*	*Cricetidae*	L: AY216519; S: AF512832
GTOV	*Sigmodon alstoni; Zygodontomys brevicauda*	*Cricetidae*	L: AY358024; S: AY129247
APOV	*Oligoryzomys mattogrossae*	*Cricetidae*	L: MF317491; S: MF317490
CHAPV	*unknown*		L: EU260464; S: EU260463
SBAV	*unknown*		L: AY358026; S: U41071
JUNV	*Calomys musculinus; Calomys callosus; Akodon azarae*	*Cricetidae*	L: AY358022; S: AY358023
MACV	*Calomys callosus*	*Cricetidae*	L: AY358021; S: AY129248
TCRV	*Amblyomma americanum (ticks); Artibeus jamaicensis*	*Ixodidae; Phyllostomidae*	L: J04340; S: M20304
LATV	*Calomys callosus*	*Cricetidae*	L: EU627612; S: AF512830
OLVV	*Necromys obscurus*	*Cricetidae*	L: AY216514; S: U34248
BCNV	*Peromyscus californicus; Neotoma macrotis*	*Cricetidae*	L: AY924390; S: AY924391
TMMV	*Sigmodon hispidus; Zygodontomys brevicauda; Oryzomys palustris*	*Cricetidae*	L: AY924393; S: AF485263
WWAV	*Neotoma albigula*	*Cricetidae*	L: AY924395; S: AF228063
ALXV	*Rodentia; Dipus sagitta*	*Dipodidae*	L: KY432892; S: KY432893
BITV	*Micaelamys namaquensis*	*Muridae*	S: MZ065537; L: MZ065536

MRWV	*Myotomys unisulcatus*	*Muridae*	L: GU078661; S: GU078660
OKAV	*Micaelamys namaquensis*	*Muridae*	L: KP867642; S: KM272988
DHWV	*Mastomys natalensis*	*Muridae*	S: MT078838; L: MT078839
GAIV	*Mastomys natalensis*	*Muridae*	L: KJ855307; S: KJ855308
KWAV	*Mus triton*	*Muridae*	S: MZ065541; L: MZ065540
MOBV	*Praomys jacksoni*	*Muridae*	L: DQ328876; S: AY342390
LUAV	*Mastomys natalensis*	*Muridae*	L: AB586645; S: AB586644
MOPV	*Mastomys natalensis*	*Muridae*	L: AY772169; S: AY772170
MORV	*Mastomys natalensis*	*Muridae*	L: EU914104; S: EU914103
LASV	*Mastomys natalensis*	*Muridae*	L: U73034; S: J04324
IPPYV	*Arvicanthis niloticus; Praomys jacksoni*	*Muridae*	L: DQ328878; S: DQ328877
KTLV	*Grammomys macmillani*	*Muridae*	L: MK935152; S: MK935153
SOLV	*Grammomys sp.*	*Muridae*	L: AB972429; S: AB972428
MRLV	*Micaelamys namaquensis*	*Muridae*	L: KP867641; S: KM272987
LIJV	*Apodemus chevrieri*	*Muridae*	L: MF414201; S: MF414202
LORV	*Bandicota sp. Bandicota indica; Niviventer fulvescens*	*Muridae*	L: KC669693; S: KC669698
WENV	*Rattus norvegicus*	*Muridae*	L: KJ909795; S: KJ909794
SOUV	*Praomys sp.*	*Muridae*	L: KP050226; S: KP050227
DANV	unknown		L: EU136039; S: EU136038
LNKV	*Mus minutoides*	*Muridae*	L: AB693151; S: AB693150
LCMV	*Mus musculus; Apodemus sylvaticus*	*Muridae*	L: AY847351; S: AY847350
LCMV	*Mus musculus; Apodemus sylvaticus*	*Muridae*	L: NC_004291.1; S: NC_004294.1
RYKV	*Mus caroli*	*Muridae*	L: KM020190; S: KM020191
LUJV	*unknown*		L: FJ952385; S: FJ952384
ROUTV	*Boa constrictor*	*Boidae*	L: KC508670; S: KC508669
UHV1	*Boa constrictor*	*Boidae*	L: KF297881; S: KF297880
TSMV2	*Boa constrictor*	*Boidae*	L: KX527582; S: KX527575
UGV1	*Boa constrictor*	*Boidae*	L: KR870022; S: KR870012
UGV2	*Boa constrictor*	*Boidae*	L: KR870029; S: KR870015
UGV3	*Boa constrictor*	*Boidae*	L: KR870023; S: KR870013
GOGV	*Boa constrictor*	*Boidae*	L: JQ717263; S: JQ717264
CASV	*Corallus annulatus*	*Boidae*	L: JQ717261; S: JQ717262
HISV1	*Boa constrictor*	*Boidae*	L: KR870031; S: KR870017
HISV2	*Boa constrictor*	*Boidae*	L: MH483042; S: MH483041
SPVV1	*Boa constrictor*	*Boidae*	L: MN567049; MN567050; S: MN567048
UnNV1	*Boa constrictor*	*Boidae*	L: MW091472; S: MW091473
BBRTV	*Neotoma albigula*	*Cricetidae*	L: EU938665; S: EF619035
CTNV	*Neotoma micropus voucher TK84773*	*Cricetidae*	S: JX237768

LULV	*Mastomys natalensis*	*Muridae*	L: AB972431; S: AB972430
MOPV	*Chlorocebus aethiops*	*Cercopithecidae*	L: AY772167; S: AY772168
SKTV	*Neotoma sp.*	*Cricetidae*	L: EU938659; S: EU123328
TTCV	*Neotoma albigula*	*Cricetidae*	L: EU938663; S: EF619034
XAPV	*Neacomys musseri*	*Cricetidae*	L: MG976577; S: MG976578

*L, M and S correspond to Large, Medium and Small segments for respective viral genomes, respectively.
